# Diaqua­bis­(benzoato-κ*O*)bis­[4,4,5,5-tetra­methyl-2-(pyridin-4-yl-κ*N*)imidazoline-1-oxyl 3-oxide]cobalt(II)

**DOI:** 10.1107/S1600536812012251

**Published:** 2012-03-31

**Authors:** Kun-Miao Wang, Jie Zhou, Fen-Hua Qian, Jing Liu

**Affiliations:** a Key Lab of Tobacco Chemistry of Yunnan, Yunnan Academy of Tobacco Science, Kunming 650106, People’s Republic of China; b School of Chemical Science and Engineering, Yunnan University, Kunming 650091, People’s Republic of China

## Abstract

The title compound, [Co(C_7_H_5_O_2_)_2_(C_12_H_16_N_3_O_2_)_2_(H_2_O)_2_], was obtained from a conventional solvent evaporation method. The complex mol­ecule is centrosymmetric, so pairs of equivalent ligands lie *trans* to each other in a slightly distorted octa­hedral CoN_2_O_4_ geometry. The Co^II^ ion is coordinated by the pyridine N atoms from NITpPy ligands [NITpPy is 4,4,5,5-tetra­methyl-2-(pyridin-4-yl)imidazoline-1-oxyl 3-oxide), water O atoms and two monodentate benzoate O atoms. The complex mol­ecules are connected by O—H⋯O hydrogen bonds between water mol­ecules and benzoate ligands, forming chains parallel to [100]. π–π stacking inter­actions between the benzoate ligands with centroid–centroid distances of 3.752 (2) Å connect the chains into layers parallel to (10-1).

## Related literature

For isotypic structures, see: Fettouhi *et al.* (1999 ▶); Zhao *et al.* (2003 ▶). For other metal nitronyl nitroxides, see: Zhou *et al.* (2006 ▶); Zhang & Zhang (2006 ▶); Zhu *et al.* (2010 ▶); Zhang *et al.* (2010 ▶).
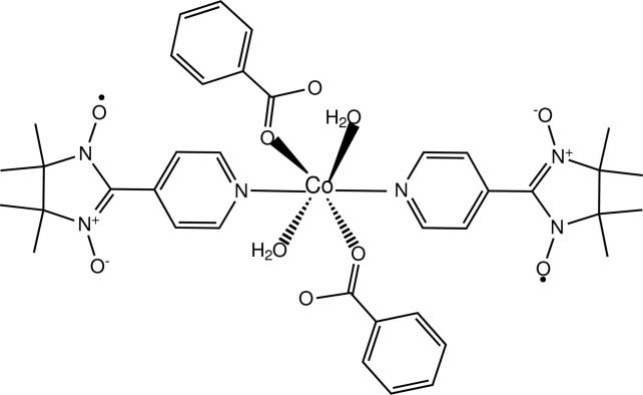


## Experimental

### 

#### Crystal data

[Co(C_7_H_5_O_2_)_2_(C_12_H_16_N_3_O_2_)_2_(H_2_O)_2_]
*M* *_r_* = 805.74
Triclinic, 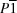

*a* = 6.778 (1) Å
*b* = 11.3381 (13) Å
*c* = 12.9109 (15) Å
α = 87.903 (2)°
β = 88.622 (2)°
γ = 79.088 (1)°
*V* = 973.5 (2) Å^3^
*Z* = 1
Mo *K*α radiation
μ = 0.51 mm^−1^
*T* = 293 K
0.49 × 0.40 × 0.38 mm

#### Data collection

Bruker APEXII 1K CCD area-detector diffractometer
Absorption correction: multi-scan (*SADABS*; Bruker, 2008 ▶) *T*_min_ = 0.790, *T*_max_ = 0.831
6131 measured reflections
4321 independent reflections
3229 reflections with *I* > 2σ(*I*)
*R*_int_ = 0.017

#### Refinement

*R*[*F* ^2^ > 2σ(*F* ^2^)] = 0.041
*wR*(*F* ^2^) = 0.115
*S* = 1.00
4321 reflections
250 parameters
2 restraints
H-atom parameters constrained
Δρ_max_ = 0.42 e Å^−3^
Δρ_min_ = −0.28 e Å^−3^

### 

Data collection: *APEX2* (Bruker, 2008 ▶); cell refinement: *SAINT* (Bruker, 2008 ▶); data reduction: *SAINT*; program(s) used to solve structure: *SHELXS97* (Sheldrick, 2008 ▶); program(s) used to refine structure: *SHELXL97* (Sheldrick, 2008 ▶); molecular graphics: *SHELXTL* (Sheldrick, 2008 ▶); software used to prepare material for publication: *SHELXTL*.

## Supplementary Material

Crystal structure: contains datablock(s) I, global. DOI: 10.1107/S1600536812012251/wm2601sup1.cif

Structure factors: contains datablock(s) I. DOI: 10.1107/S1600536812012251/wm2601Isup2.hkl

Additional supplementary materials:  crystallographic information; 3D view; checkCIF report

## Figures and Tables

**Table 1:** Selected bond lengths (Å)

Co1—O2	2.0586 (15)
Co1—O5	2.1521 (13)
Co1—N1	2.1743 (16)

**Table 2:** Hydrogen-bond geometry (Å, °)

*D*—H⋯*A*	*D*—H	H⋯*A*	*D*⋯*A*	*D*—H⋯*A*
O5—H5*B*⋯O1^i^	0.87	2.06	2.857 (2)	152
O5—H5*C*⋯O1^ii^	0.88	1.78	2.635 (2)	165

Symmetry codes: (i) 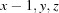; (ii) 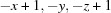.

**Fig. 1.**
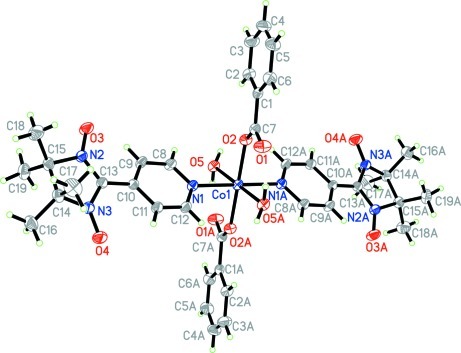
The molecular structure of the title complex with the atom-numbering scheme (symmetry code: A -x + 1, -y, -z + 1). Ellipsoids were drawn at the 30% probability level.

**Fig. 2.**
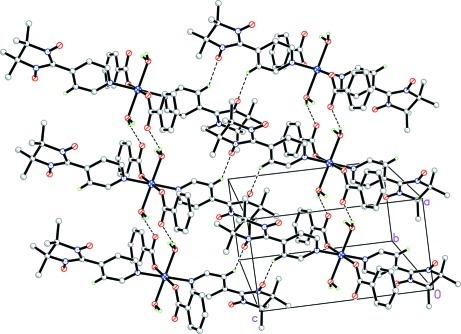
The chain structure formed by O–H···O hydrogen bonds (dotted lines).

**Fig. 3.**
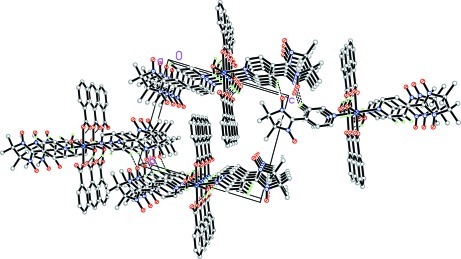
The packing of the title compound.

## supplementary crystallographic information

### Comment

In recent years, nitronyl nitroxides were widely used in the design and synthesis of new molecule-based magnetic materials because of their ability to act as magnetic couplers. Various metal nitronyl nitroxides complexes have been reported (Zhou *et al.*, 2006; Zhang & Zhang 2006; Zhu *et al.*, 2010; Zhang *et al.* 2010). In this paper, we report the Co^II^ complex with NITpPy (NITpPy is 2-(4-pyridyl-κN)-4,4,5,5-tetramethylimidazoline-1-oxyl 3-oxide, nitronyl nitroxide) and an additional benzoate anion.

The title compound is isotypic with the analogous Mn^II^ (Fettouhi *et al.*, 1999) and Ni^II^ structures (Zhao *et al.*, 2003). It is a centrosymmetric monoculear compound (Fig. 1). The Co^II^ ion is located on a crystallographic center of inversion and adopts a slightly distorted octahedral coordination environment defined by two pyridine N atoms from the NITpPy ligands [Co—N = 2.1743 (17) Å] and four oxygen atoms from carboxylate anions [Co—O = 2.0590 (15) Å] and water molecules [Co—O = 2.1521 (13) Å]. The benzoate ligands exhibit a mondentate coordination mode. The dihedral angle between the mean-planes of the benzoate and the pyridine-imidazolin rings is 76.97 (8) °. In the crystal structure, O—H···O hydrogen-bonding interactions between water molecules and benzoate anions are observed which join the mononuclear units into a chain structure along [100] (Fig. 2, Table 2). Furthermore, π—π stacking interactions among the benzoate aromatic rings with a *Cg*···*Cg* distance of 3.752 (2) Å connect neighbouring chains into a layered structure parallel to (101) (Fig. 3).

### Experimental

The title compound was prepared by dropwise adding a methanol solution (15 ml) of cobalt(II) benzoate (0.0067 g, 0.2 mmol) to a solution of NITpPy (0.0468 g, 0.2 mmol) in dichloromethane (10 ml). The mixture was then stirred for 0.5 h and filtered. The clear blue filtrate was kept in the dark and was slowly evaporated at room temperature. A few weeks later, dark-blue crystals suitable for X-ray analysis were obtained.

### Refinement

The H atoms of aromatic ring and methyl groups were generated geometrically and were included in the refinement in the riding model approximationwith *d*(C—H) = 0.93 Å, *U*_iso_= 1.2 *U*_eq_(C) and *d*(C—H) = 0.96 Å, *U*_iso_= 1.5 *U*_eq_(C), respectively. The H atoms of water molecules were located in difference Fourier maps and were constrained with *d*(O—H) =0.88 Å, *U*_iso_= 1.2 *U*_eq_(O).

### Crystal data

**Table:**

[Co(C_7_H_5_O_2_)_2_(C_12_H_16_N_3_O_2_)_2_(H_2_O)_2_]	*Z* = 1
*M**_r_* = 805.74	*F*(000) = 423
Triclinic, *P*1	*D*_x_ = 1.374 Mg m^−^^3^
Hall symbol: -P 1	Mo *K*α radiation, λ = 0.71073 Å
*a* = 6.778 (1) Å	Cell parameters from 254 reflections
*b* = 11.3381 (13) Å	θ = 1.6–25.0°
*c* = 12.9109 (15) Å	µ = 0.51 mm^−^^1^
α = 87.903 (2)°	*T* = 293 K
β = 88.622 (2)°	Block, dark-blue
γ = 79.088 (1)°	0.49 × 0.40 × 0.38 mm
*V* = 973.5 (2) Å^3^	

### Data collection

**Table:**

Bruker APEXII 1K CCD area-detector diffractometer	4321 independent reflections
Radiation source: fine-focus sealed tube	3229 reflections with *I* > 2σ(*I*)
Graphite monochromator	*R*_int_ = 0.017
phi and ω scans	θ_max_ = 28.4°, θ_min_ = 1.6°
Absorption correction: multi-scan (*SADABS*; Bruker, 2008)	*h* = −6→8
*T*_min_ = 0.790, *T*_max_ = 0.831	*k* = −9→14
6131 measured reflections	*l* = −16→16

### Refinement

**Table:**

Refinement on *F*^2^	Primary atom site location: structure-invariant direct methods
Least-squares matrix: full	Secondary atom site location: difference Fourier map
*R*[*F*^2^ > 2σ(*F*^2^)] = 0.041	Hydrogen site location: inferred from neighbouring sites
*wR*(*F*^2^) = 0.115	H-atom parameters constrained
*S* = 1.00	*w* = 1/[σ^2^(*F*_o_^2^) + (0.0633*P*)^2^ + 0.1038*P*] where *P* = (*F*_o_^2^ + 2*F*_c_^2^)/3
4321 reflections	(Δ/σ)_max_ < 0.001
250 parameters	Δρ_max_ = 0.42 e Å^−^^3^
2 restraints	Δρ_min_ = −0.28 e Å^−^^3^

### Special details

**Table:**

Geometry. All e.s.d.'s (except the e.s.d. in the dihedral angle between two l.s. planes) are estimated using the full covariance matrix. The cell e.s.d.'s are taken into account individually in the estimation of e.s.d.'s in distances, angles and torsion angles; correlations between e.s.d.'s in cell parameters are only used when they are defined by crystal symmetry. An approximate (isotropic) treatment of cell e.s.d.'s is used for estimating e.s.d.'s involving l.s. planes.
Refinement. Refinement of *F*^2^ against ALL reflections. The weighted *R*-factor *wR* and goodness of fit *S* are based on *F*^2^, conventional *R*-factors *R* are based on *F*, with *F* set to zero for negative *F*^2^. The threshold expression of *F*^2^ > σ(*F*^2^) is used only for calculating *R*-factors(gt) *etc*. and is not relevant to the choice of reflections for refinement. *R*-factors based on *F*^2^ are statistically about twice as large as those based on *F*, and *R*- factors based on ALL data will be even larger.

### Fractional atomic coordinates and isotropic or equivalent isotropic displacement parameters (Å^2^)

**Table:**

	*x*	*y*	*z*	*U*_iso_*/*U*_eq_	
Co1	0.5000	0.0000	0.5000	0.03209 (14)	
O1	0.8445 (2)	0.17316 (15)	0.51875 (14)	0.0559 (5)	
O2	0.5208 (2)	0.16870 (13)	0.54704 (11)	0.0397 (4)	
O3	0.7545 (3)	−0.06107 (16)	1.04069 (13)	0.0615 (5)	
O4	1.1308 (3)	−0.38091 (16)	0.87086 (14)	0.0674 (6)	
O5	0.2113 (2)	0.01876 (14)	0.57722 (11)	0.0397 (4)	
H5B	0.1162	0.0824	0.5727	0.048*	
H5C	0.1736	−0.0444	0.5524	0.048*	
N1	0.6275 (2)	−0.07874 (15)	0.64531 (12)	0.0346 (4)	
N2	0.8799 (3)	−0.15629 (16)	1.02104 (13)	0.0392 (4)	
N3	1.0640 (3)	−0.30529 (16)	0.93918 (14)	0.0405 (4)	
C1	0.6393 (3)	0.34249 (19)	0.59181 (15)	0.0364 (5)	
C2	0.4538 (4)	0.3959 (2)	0.63003 (18)	0.0470 (6)	
H2A	0.3473	0.3549	0.6300	0.056*	
C3	0.4246 (5)	0.5105 (2)	0.6686 (2)	0.0624 (7)	
H3A	0.2992	0.5457	0.6950	0.075*	
C4	0.5802 (5)	0.5721 (2)	0.6680 (2)	0.0647 (8)	
H4A	0.5605	0.6492	0.6933	0.078*	
C5	0.7648 (5)	0.5196 (2)	0.6299 (2)	0.0630 (7)	
H5A	0.8704	0.5614	0.6296	0.076*	
C6	0.7960 (4)	0.4054 (2)	0.59202 (19)	0.0504 (6)	
H6A	0.9222	0.3705	0.5666	0.060*	
C7	0.6710 (3)	0.21875 (19)	0.54954 (15)	0.0359 (5)	
C8	0.5444 (3)	−0.0455 (2)	0.73736 (16)	0.0405 (5)	
H8A	0.4223	0.0082	0.7381	0.049*	
C9	0.6290 (3)	−0.0863 (2)	0.83080 (16)	0.0401 (5)	
H9A	0.5647	−0.0603	0.8926	0.048*	
C10	0.8118 (3)	−0.16670 (18)	0.83235 (15)	0.0334 (4)	
C11	0.8974 (3)	−0.2025 (2)	0.73650 (16)	0.0413 (5)	
H11A	1.0189	−0.2565	0.7330	0.050*	
C12	0.8007 (3)	−0.1570 (2)	0.64743 (16)	0.0407 (5)	
H12A	0.8599	−0.1827	0.5844	0.049*	
C13	0.9138 (3)	−0.20920 (19)	0.92853 (16)	0.0355 (5)	
C14	1.1671 (3)	−0.3070 (2)	1.04120 (17)	0.0420 (5)	
C15	1.0002 (3)	−0.2294 (2)	1.10556 (16)	0.0416 (5)	
C16	1.2326 (4)	−0.4350 (2)	1.0811 (2)	0.0634 (8)	
H16A	1.3368	−0.4761	1.0365	0.095*	
H16B	1.1200	−0.4753	1.0821	0.095*	
H16C	1.2825	−0.4351	1.1501	0.095*	
C17	1.3493 (4)	−0.2487 (3)	1.0178 (3)	0.0697 (8)	
H17A	1.3050	−0.1674	0.9928	0.105*	
H17B	1.4329	−0.2931	0.9659	0.105*	
H17C	1.4245	−0.2489	1.0798	0.105*	
C18	1.0723 (4)	−0.1453 (3)	1.1797 (2)	0.0671 (8)	
H18A	0.9593	−0.1020	1.2174	0.101*	
H18B	1.1378	−0.0894	1.1409	0.101*	
H18C	1.1652	−0.1915	1.2275	0.101*	
C19	0.8581 (4)	−0.3005 (3)	1.16147 (19)	0.0585 (7)	
H19A	0.7591	−0.2468	1.2002	0.088*	
H19B	0.9331	−0.3595	1.2079	0.088*	
H19C	0.7926	−0.3399	1.1116	0.088*	

### Atomic displacement parameters (Å^2^)

**Table:**

	*U*^11^	*U*^22^	*U*^33^	*U*^12^	*U*^13^	*U*^23^
Co1	0.0273 (2)	0.0394 (2)	0.0296 (2)	−0.00598 (16)	−0.00111 (15)	−0.00383 (16)
O1	0.0318 (8)	0.0554 (10)	0.0813 (13)	−0.0077 (7)	0.0055 (8)	−0.0217 (9)
O2	0.0333 (8)	0.0419 (9)	0.0447 (8)	−0.0078 (7)	0.0001 (6)	−0.0080 (7)
O3	0.0680 (12)	0.0605 (11)	0.0425 (9)	0.0255 (9)	−0.0084 (8)	−0.0143 (8)
O4	0.0809 (13)	0.0557 (11)	0.0514 (10)	0.0248 (10)	0.0013 (9)	−0.0096 (9)
O5	0.0299 (7)	0.0490 (9)	0.0401 (8)	−0.0064 (7)	−0.0006 (6)	−0.0062 (7)
N1	0.0316 (9)	0.0404 (10)	0.0315 (9)	−0.0053 (7)	−0.0008 (7)	−0.0033 (8)
N2	0.0387 (10)	0.0420 (10)	0.0329 (9)	0.0033 (8)	−0.0057 (7)	−0.0032 (8)
N3	0.0386 (10)	0.0408 (10)	0.0369 (10)	0.0048 (8)	0.0013 (8)	−0.0006 (8)
C1	0.0415 (12)	0.0394 (12)	0.0280 (10)	−0.0067 (9)	−0.0050 (9)	−0.0002 (9)
C2	0.0470 (13)	0.0466 (14)	0.0472 (13)	−0.0073 (11)	0.0019 (11)	−0.0068 (11)
C3	0.0673 (18)	0.0538 (16)	0.0616 (17)	0.0022 (14)	0.0043 (14)	−0.0155 (13)
C4	0.095 (2)	0.0406 (14)	0.0594 (17)	−0.0117 (15)	−0.0153 (16)	−0.0090 (13)
C5	0.078 (2)	0.0513 (16)	0.0669 (18)	−0.0283 (15)	−0.0089 (15)	−0.0052 (14)
C6	0.0482 (14)	0.0533 (15)	0.0523 (14)	−0.0157 (12)	−0.0025 (11)	−0.0038 (12)
C7	0.0334 (11)	0.0412 (12)	0.0327 (10)	−0.0053 (9)	−0.0049 (9)	−0.0012 (9)
C8	0.0297 (11)	0.0506 (13)	0.0376 (11)	0.0030 (9)	−0.0013 (9)	−0.0065 (10)
C9	0.0333 (11)	0.0535 (14)	0.0303 (10)	0.0004 (10)	0.0029 (9)	−0.0072 (10)
C10	0.0343 (11)	0.0343 (11)	0.0309 (10)	−0.0044 (9)	0.0000 (8)	−0.0016 (8)
C11	0.0364 (11)	0.0434 (13)	0.0389 (12)	0.0052 (10)	0.0026 (9)	−0.0027 (10)
C12	0.0427 (12)	0.0451 (13)	0.0310 (10)	−0.0002 (10)	0.0050 (9)	−0.0034 (9)
C13	0.0320 (10)	0.0373 (11)	0.0353 (11)	−0.0016 (9)	−0.0007 (8)	−0.0020 (9)
C14	0.0335 (11)	0.0457 (13)	0.0444 (12)	−0.0020 (9)	−0.0075 (9)	0.0049 (10)
C15	0.0389 (12)	0.0477 (13)	0.0360 (11)	−0.0025 (10)	−0.0103 (9)	0.0030 (10)
C16	0.0605 (17)	0.0591 (17)	0.0615 (17)	0.0094 (13)	−0.0072 (13)	0.0130 (14)
C17	0.0371 (14)	0.078 (2)	0.094 (2)	−0.0118 (13)	0.0003 (14)	0.0059 (17)
C18	0.0735 (19)	0.0720 (19)	0.0549 (16)	−0.0068 (15)	−0.0233 (14)	−0.0131 (14)
C19	0.0482 (14)	0.0751 (19)	0.0479 (14)	−0.0038 (13)	0.0041 (11)	0.0131 (13)

### Geometric parameters (Å, º)

**Table:**

Co1—O2	2.0586 (15)	C5—H5A	0.9300
Co1—O2^i^	2.0586 (15)	C6—H6A	0.9300
Co1—O5	2.1521 (13)	C8—C9	1.375 (3)
Co1—O5^i^	2.1521 (13)	C8—H8A	0.9300
Co1—N1^i^	2.1743 (16)	C9—C10	1.392 (3)
Co1—N1	2.1743 (16)	C9—H9A	0.9300
O1—C7	1.253 (3)	C10—C11	1.395 (3)
O2—C7	1.258 (2)	C10—C13	1.457 (3)
O3—N2	1.271 (2)	C11—C12	1.372 (3)
O4—N3	1.268 (2)	C11—H11A	0.9300
O5—H5B	0.8731	C12—H12A	0.9300
O5—H5C	0.8777	C14—C16	1.511 (3)
N1—C12	1.331 (3)	C14—C17	1.527 (3)
N1—C8	1.339 (2)	C14—C15	1.541 (3)
N2—C13	1.351 (3)	C15—C19	1.522 (3)
N2—C15	1.504 (3)	C15—C18	1.527 (3)
N3—C13	1.348 (2)	C16—H16A	0.9600
N3—C14	1.503 (3)	C16—H16B	0.9600
C1—C2	1.377 (3)	C16—H16C	0.9600
C1—C6	1.387 (3)	C17—H17A	0.9600
C1—C7	1.499 (3)	C17—H17B	0.9600
C2—C3	1.386 (3)	C17—H17C	0.9600
C2—H2A	0.9300	C18—H18A	0.9600
C3—C4	1.370 (4)	C18—H18B	0.9600
C3—H3A	0.9300	C18—H18C	0.9600
C4—C5	1.368 (4)	C19—H19A	0.9600
C4—H4A	0.9300	C19—H19B	0.9600
C5—C6	1.378 (4)	C19—H19C	0.9600
			
O2—Co1—O2^i^	180.00 (2)	C8—C9—C10	119.56 (18)
O2—Co1—O5	88.92 (6)	C8—C9—H9A	120.2
O2^i^—Co1—O5	91.08 (6)	C10—C9—H9A	120.2
O2—Co1—O5^i^	91.08 (6)	C9—C10—C11	116.72 (19)
O2^i^—Co1—O5^i^	88.92 (6)	C9—C10—C13	122.29 (18)
O5—Co1—O5^i^	180.0	C11—C10—C13	120.96 (18)
O2—Co1—N1^i^	89.66 (6)	C12—C11—C10	119.33 (19)
O2^i^—Co1—N1^i^	90.34 (6)	C12—C11—H11A	120.3
O5—Co1—N1^i^	93.30 (6)	C10—C11—H11A	120.3
O5^i^—Co1—N1^i^	86.70 (6)	N1—C12—C11	124.29 (19)
O2—Co1—N1	90.34 (6)	N1—C12—H12A	117.9
O2^i^—Co1—N1	89.66 (6)	C11—C12—H12A	117.9
O5—Co1—N1	86.70 (6)	N3—C13—N2	108.43 (18)
O5^i^—Co1—N1	93.30 (6)	N3—C13—C10	125.64 (19)
N1^i^—Co1—N1	180.00 (4)	N2—C13—C10	125.87 (18)
C7—O2—Co1	129.93 (13)	N3—C14—C16	110.0 (2)
Co1—O5—H5B	124.9	N3—C14—C17	105.2 (2)
Co1—O5—H5C	98.0	C16—C14—C17	110.6 (2)
H5B—O5—H5C	111.6	N3—C14—C15	100.66 (16)
C12—N1—C8	116.32 (18)	C16—C14—C15	115.4 (2)
C12—N1—Co1	121.37 (13)	C17—C14—C15	114.1 (2)
C8—N1—Co1	122.16 (14)	N2—C15—C19	106.03 (18)
O3—N2—C13	126.83 (17)	N2—C15—C18	109.42 (19)
O3—N2—C15	121.29 (17)	C19—C15—C18	110.5 (2)
C13—N2—C15	111.73 (17)	N2—C15—C14	100.80 (16)
O4—N3—C13	126.87 (19)	C19—C15—C14	114.0 (2)
O4—N3—C14	121.14 (17)	C18—C15—C14	115.2 (2)
C13—N3—C14	111.63 (18)	C14—C16—H16A	109.5
C2—C1—C6	118.9 (2)	C14—C16—H16B	109.5
C2—C1—C7	120.6 (2)	H16A—C16—H16B	109.5
C6—C1—C7	120.5 (2)	C14—C16—H16C	109.5
C1—C2—C3	120.5 (2)	H16A—C16—H16C	109.5
C1—C2—H2A	119.8	H16B—C16—H16C	109.5
C3—C2—H2A	119.8	C14—C17—H17A	109.5
C4—C3—C2	120.1 (3)	C14—C17—H17B	109.5
C4—C3—H3A	120.0	H17A—C17—H17B	109.5
C2—C3—H3A	120.0	C14—C17—H17C	109.5
C5—C4—C3	119.8 (3)	H17A—C17—H17C	109.5
C5—C4—H4A	120.1	H17B—C17—H17C	109.5
C3—C4—H4A	120.1	C15—C18—H18A	109.5
C4—C5—C6	120.7 (3)	C15—C18—H18B	109.5
C4—C5—H5A	119.7	H18A—C18—H18B	109.5
C6—C5—H5A	119.7	C15—C18—H18C	109.5
C5—C6—C1	120.1 (2)	H18A—C18—H18C	109.5
C5—C6—H6A	120.0	H18B—C18—H18C	109.5
C1—C6—H6A	120.0	C15—C19—H19A	109.5
O1—C7—O2	124.8 (2)	C15—C19—H19B	109.5
O1—C7—C1	117.8 (2)	H19A—C19—H19B	109.5
O2—C7—C1	117.39 (18)	C15—C19—H19C	109.5
N1—C8—C9	123.77 (19)	H19A—C19—H19C	109.5
N1—C8—H8A	118.1	H19B—C19—H19C	109.5
C9—C8—H8A	118.1		
			
O5—Co1—O2—C7	164.30 (17)	C10—C11—C12—N1	0.5 (4)
O5^i^—Co1—O2—C7	−15.70 (17)	O4—N3—C13—N2	176.0 (2)
N1^i^—Co1—O2—C7	−102.40 (17)	C14—N3—C13—N2	−10.9 (2)
N1—Co1—O2—C7	77.60 (17)	O4—N3—C13—C10	−6.6 (4)
O2—Co1—N1—C12	−118.25 (17)	C14—N3—C13—C10	166.5 (2)
O2^i^—Co1—N1—C12	61.75 (17)	O3—N2—C13—N3	177.5 (2)
O5—Co1—N1—C12	152.85 (17)	C15—N2—C13—N3	−7.0 (2)
O5^i^—Co1—N1—C12	−27.15 (17)	O3—N2—C13—C10	0.0 (4)
O2—Co1—N1—C8	57.16 (17)	C15—N2—C13—C10	175.55 (19)
O2^i^—Co1—N1—C8	−122.84 (17)	C9—C10—C13—N3	164.2 (2)
O5—Co1—N1—C8	−31.73 (17)	C11—C10—C13—N3	−17.9 (3)
O5^i^—Co1—N1—C8	148.27 (17)	C9—C10—C13—N2	−18.8 (3)
C6—C1—C2—C3	−0.4 (3)	C11—C10—C13—N2	159.2 (2)
C7—C1—C2—C3	−179.7 (2)	O4—N3—C14—C16	−41.2 (3)
C1—C2—C3—C4	0.7 (4)	C13—N3—C14—C16	145.3 (2)
C2—C3—C4—C5	−0.5 (4)	O4—N3—C14—C17	77.9 (3)
C3—C4—C5—C6	0.1 (4)	C13—N3—C14—C17	−95.7 (2)
C4—C5—C6—C1	0.3 (4)	O4—N3—C14—C15	−163.4 (2)
C2—C1—C6—C5	−0.1 (3)	C13—N3—C14—C15	23.1 (2)
C7—C1—C6—C5	179.2 (2)	O3—N2—C15—C19	77.5 (3)
Co1—O2—C7—O1	2.6 (3)	C13—N2—C15—C19	−98.3 (2)
Co1—O2—C7—C1	−177.94 (12)	O3—N2—C15—C18	−41.6 (3)
C2—C1—C7—O1	−177.8 (2)	C13—N2—C15—C18	142.5 (2)
C6—C1—C7—O1	2.8 (3)	O3—N2—C15—C14	−163.4 (2)
C2—C1—C7—O2	2.6 (3)	C13—N2—C15—C14	20.7 (2)
C6—C1—C7—O2	−176.73 (19)	N3—C14—C15—N2	−24.2 (2)
C12—N1—C8—C9	0.9 (3)	C16—C14—C15—N2	−142.5 (2)
Co1—N1—C8—C9	−174.70 (17)	C17—C14—C15—N2	87.9 (2)
N1—C8—C9—C10	0.1 (4)	N3—C14—C15—C19	88.9 (2)
C8—C9—C10—C11	−0.8 (3)	C16—C14—C15—C19	−29.4 (3)
C8—C9—C10—C13	177.2 (2)	C17—C14—C15—C19	−159.0 (2)
C9—C10—C11—C12	0.5 (3)	N3—C14—C15—C18	−141.8 (2)
C13—C10—C11—C12	−177.5 (2)	C16—C14—C15—C18	99.9 (3)
C8—N1—C12—C11	−1.2 (3)	C17—C14—C15—C18	−29.8 (3)
Co1—N1—C12—C11	174.42 (18)		

Symmetry code: (i) −*x*+1, −*y*, −*z*+1.

### Hydrogen-bond geometry (Å, º)

**Table:**

*D*—H···*A*	*D*—H	H···*A*	*D*···*A*	*D*—H···*A*
O5—H5*B*···O1^ii^	0.87	2.06	2.857 (2)	152
O5—H5*C*···O1^i^	0.88	1.78	2.635 (2)	165

Symmetry codes: (i) −*x*+1, −*y*, −*z*+1; (ii) *x*−1, *y*, *z*.
